# Exploring extended [^18^F]FDG kinetics in lymphoma with ultra-late LAFOV-PET/CT

**DOI:** 10.1007/s00259-026-07774-w

**Published:** 2026-02-11

**Authors:** Matthias Weissinger, Stephan Ursprung, Johann Jacoby, Jonas Vogel, Eduardo Calderón, Brigitte Gückel, Fabian Schmidt, Johannes Schwenck, Helmut Dittmann, Konstantin Nikolaou, Christian la Fougère, Christian Philipp Reinert

**Affiliations:** 1 Nuclear Medicine and Clinical Molecular Imaging, Department of Radiology, University Hospital of Tuebingen, Otfried-Mueller-Strasse 14, 72076 Tuebingen, Germany; 2 Diagnostic and Interventional Radiology, University Department of Radiology, University Hospital of Tuebingen, Hoppe-Seyler-Str. 3, 72076 Tuebingen, Germany; 3 MVZ Nuclear Medicine, Marienhospital Stuttgart, Boeheimstraße 37, 70199 Stuttgart, Germany; 4 Institute for Clinical Epidemiology and Applied Biometry, University Hospital Tuebingen, Silcherstr. 5, 72076 Tuebingen, Germany; 5 Department of Radiology and Nuclear Medicine, ViDia Hospital, Suedendstr. 32, 76135 Karlsruhe, Germany; 6 Department of Preclinical Imaging and Radiopharmacy, Werner Siemens Imaging Center, Eberhard Karls University Tuebingen, Roentgenweg 13, 72076 Tübingen, Germany; 7 Cluster of Excellence iFIT (EXC 2180) “Image Guided and Functionally Instructed Tumor Therapies”, University of Tübingen, Tübingen, Germany; 8 German Cancer Consortium (DKTK), Partner Site Tübingen, Tübingen, Germany

**Keywords:** Delayed LAFOV PET, Ultra-late PET, [^18^F]FDG efflux, [^18^F]FDG kinetic, Multi Time-Point kinetic, Glucose-6-phosphatase-β activity, [^18^F]FDG trapping

## Abstract

**Purpose:**

This prospective trial investigated extended [^18^F]FDG kinetics in lymphoma to provide in-vivo insights into glucose metabolism with potential relevance for staging and risk stratification.

**Methods:**

Fifteen consecutive, treatment- naïve lymphoma patients (4 Hodgkin, 11 non-Hodgkin) underwent routine whole-body [^18^F]FDG-PET/CT at 1 h post injection (p.i., injected activity 3.02 ± 0.34 MBq/kg) followed by additional Long Axial Field-Of-View (LAFOV)-PET/CT scans at 3 h and 6 h p.i. (Biograph Vision Quadra^®^, Siemens Healthineers; acquisition 5/15/30 min). Standardised uptake values (SUV) of lymphoma, benign lymph nodes, organs and reference tissues were quantified and multi time-point kinetics were described using Retention Indices (RI) and linear/quadratic trajectory analyses. Image quality was rated by two blinded readers on a 5-point Likert scale.

**Results:**

Image quality remained diagnostic in all datasets. Median Tumour-to-Background Ratio (TBR) increased significantly from 4.1 (1 h p.i.) to 12.5 (3 h p.i.) and 23.9 (6 h p.i.), *p* < 0.001. High-grade lymphoma exhibited an almost linear SUV rise, whereas low-grade entities followed a parabolic course, peaking at 3 h p.i. Benign lymph nodes demonstrated constant uptake (1 h: 0.9 ± 0.3, 3 h: 0.8 ± 0.5, 6 h: 0.8 ± 0.4). RIs showed a significant increase in [^18^F]FDG uptake over time in lymphoma, compared with a decline in benign lymph nodes (1–3 h p.i.: 19.4% vs. -14.4%, *p* < 0.001).

**Conclusion:**

The LAFOV scanner enables high-quality [^18^F]FDG PET imaging for up to 6 h p.i., with a six-fold increase of TBR in ultra-late scans 6 h p.i. Extended [^18^F]FDG kinetic analysis differentiates high- and low-grade lymphomas from benign lymph nodes and reveals a significant decline in tracer uptake in low-grade lymphomas between 3 and 6 h p.i.

**Trial registration:**

DRKS00027307. Registered 26 November 2021.

**Supplementary Information:**

The online version contains supplementary material available at 10.1007/s00259-026-07774-w.

## Introduction

Positron emission tomography (PET) using [^18^F]fluorodeoxyglucose ([^18^F]FDG) has become the method of choice in the staging and therapeutic response assessment of malignant lymphoma [1–8]. International guidelines recommend [^18^F]FDG PET/CT for staging before, during and after therapy for most lymphoma [3, 6, 7, 9, 10], except for subtypes with typically low or heterogeneous [^18^F]FDG avidity such as marginal zone lymphoma, small lymphocytic lymphoma/chronic lymphocytic leukaemia (SLL/CLL), lymphoplasmacytic lymphoma (including Waldenström macroglobulinaemia) and certain cutaneous T-cell lymphoma like Mycosis fungoides [8, 11].

Conventionally, PET scans are performed 1 h after [^18^F]FDG injection (p.i.) [12]. This practice, which was implemented in the Lugano classification guidelines by the International Conference on Malignant Lymphoma [1, 2], is based on the [^18^F]FDG avidity of many lymphoma subtypes, which has been extensively studied by Weiler-Sagie et al. [13]. Lymphoma represent a heterogeneous group of lymphoproliferative disorders showing considerable variability in [^18^F]FDG uptake and kinetics [3, 5–8, 14]. For [^18^F]FDG-avid lymphoma, identifying pathological uptake usually requires a comparison of lesion activity with that of the blood pool or liver parenchyma [2, 3, 6, 7]. However, due to the non-specific nature of [^18^F]FDG uptake, distinguishing between malignant and inflammatory uptake remains a significant challenge in clinical practice.

Emerging evidence suggests that delayed [^18^F]FDG imaging could offer diagnostic advantages [14–17]. Previous studies indicate an increasing [^18^F]FDG uptake in lymphoma and solid tumours beyond the conventional examination window 1 h p.i., while background organ activity continues to decline [14, 18, 19]. Delayed imaging at 2 h p.i. may even improve the detectability of lesions and differentiate between malignant and benign lymph nodes [14, 19]. Additionally, dual time-point and dynamic PET imaging were shown to be at least as accurate as the Standardised Uptake Value (SUV) in detecting lymphoma and lymph node metastases [14, 20–24].

Furthermore, data on [^18^F]FDG kinetics acquired in delayed PET images may provide additional information for distinguishing Hodgkin lymphoma from indolent non-Hodgkin lymphoma, as well as for differentiating between indolent and aggressive non-Hodgkin lymphoma [9]. Despite these benefits, the short half-life of fluorine-18 results in a considerable decline in count rates over time, effectively limiting whole-body PET imaging with conventional scanners within about 2 h p.i. Recent advances in PET technology, particularly Long Axial Field-Of-View (LAFOV) PET systems with high timing resolution, provide markedly increased sensitivity, enabling PET imaging under low-count conditions potentially overcoming this limitation [25–28].

Our study aims to investigate the extended pharmacokinetics of [^18^F]FDG in lymphoma using serial [^18^F]FDG-PET scans with a LAFOV PET/CT scanner and ultra-late PET scans up to 6 h p.i. and to evaluate their diagnostic value in the detection and differentiation of lymphoma subtypes.

## Materials and methods

### Study design and ethics

This single‑centre investigator‑initiated trial adhered to the Declaration of Helsinki and German radiation‑protection regulations. The study was approved by the local ethics committee (registry no. 704/2021BO1). Written informed consent was obtained from all patients for the use of pseudonymised data for research purposes.

### Patient cohort

Overall 19 patients with an initial diagnosis of lymphoma (Hodgkin’s disease and non-Hodgkin) and a clinical indication for [^18^F]FDG PET/CT for primary staging and treatment planning were enrolled in the single-centre prospective trial.

**Fig. 1:**
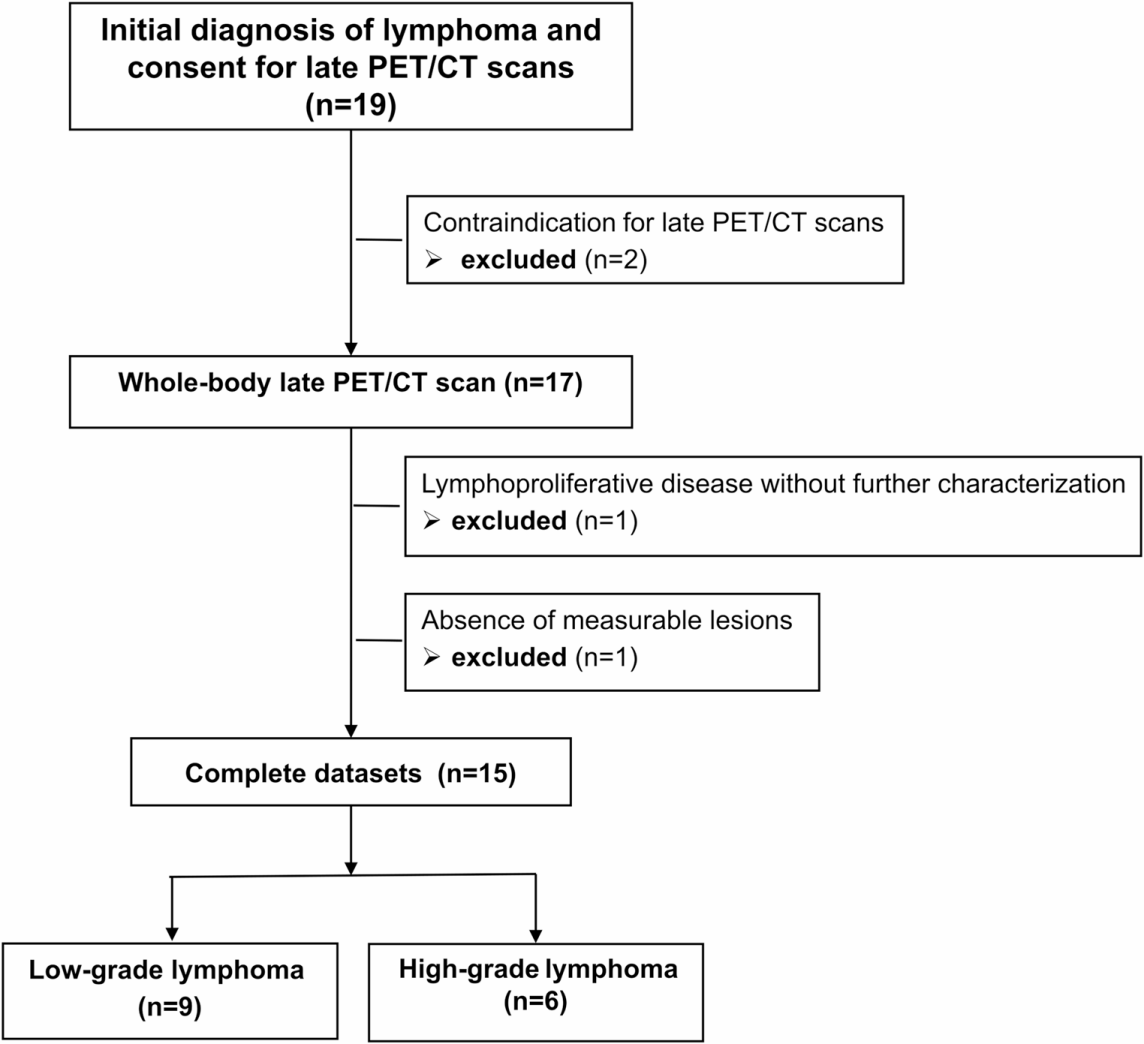
Consort flow chart. Four patients were excluded: two were not fit to complete late scans, one lacked conclusive histology, and one had no measurable lesion after excisional biopsy

For inclusion, participants had to be capable of accepting the pseudonymised prospective data collection and tolerate a waiting time of 3 to 5 h between the regular and study-specific [^18^F]FDG-PET/CT scans. Patients’ age had to be ≥ 18 years and written consent from the patient was required. Exclusion criteria encompassed pregnancy and breastfeeding, claustrophobia, reduced performance status and/or discomfort/pain while lying on their back, making it potentially challenging to tolerate waiting and additional scanning time. Additional exclusion criteria included conditions stipulated by the Radiation Protection Ordinance (StrlSchV), as well as limited capacity to provide informed consent. A total of 19 patients were recruited for the study and 15 patients were included in the final evaluation, as shown in detail in the CONSORT flow chart in Fig. 1.

### PET/CT scanner

All measurements were conducted using a state-of-the-art LAFOV PET/CT system (Biograph Vision Quadra^®^, Siemens Healthineers). This system employs 3.2 × 3.2 × 20 mm^3^ Lutetium Oxyorthosilicate (LSO) crystals coupled to Silicon Photomultipliers (SiPMs) and provides a Time-of-Flight (TOF) resolution of 228 ps. The detectors are arranged cylindrically, featuring a diameter of 82 cm and 106 cm axial length [26]. Data acquisitions were performed employing a Maximum Ring Difference (MRD) setting of 85 for which the system’s total sensitivity, as per NEMA NU 2–2018, is 83 kcps/MBq, maintaining a consistent sensitivity profile of approximately 200 cps/MBq/plane across the central 80 cm of the axial field of view [26].

### Study protocol

All patients fasted for at least 6 h prior to intravenous administration and refrained from using long-acting insulin. Blood glucose levels were measured before injection and had to be below 160 mg/dL. The [^18^F]FDG activity was calculated based on the subject’s body weight with an activity of 3 MBq per kg. The clinical PET/CT scan was performed using a care dose protocol with a maximum of 120 kV (aluminium and tin filter) and weight adjusted mAs modulation. A weight-adjusted dose of CT contrast agent (Ultravist 370^®^) was administered unless contraindicated. Study-related late [^18^F]FDG PET/CT scans were performed at 3 h p.i. and ultra-late scans at 6 h p.i., in addition to the routine PET/CT scan 1 h p.i. Patients were free to eat and go for a walk after the first scan. To match the image quality of the routine 5-min PET scan at 1 h p.i., decay-adapted acquisition times of 15 min at 3 h and 30 min at 6 h p.i. scan were applied. Images were reconstructed with an Ordinary Poisson Ordered Subset Expectation Maximization (OP-OSEM) algorithm with 4 iterations and 5 subsets with point spread function modelling and with TOF information. The image matrix size was 440 × 440 × 645 with a voxel size of 1.65 × 1.65 × 1.65 mm^3^. PET attenuation correction for late scans was achieved using an ultra-low-dose CT protocol with a tube current of 6 mAs and a tube voltage of 140 kV with a tin filter.

### Quantitative image analysis

PET datasets for all patients were systematically evaluated at three time points: 1 h, 3 h, and 6 h p.i. Each evaluation involved precise placement of standardised Volumes of Interest (VOI) within target tissues from the initial PET scan 1 h p.i. To ensure consistency across time points, VOIs were transferred to the corresponding anatomical locations on the 3 h and 6 h p.i. scans, with isocontours within each VOI determined individually for every time point. Lymphoma lesions and benign lymph nodes were defined based on biopsy results and/or imaging criteria [29].

The measurements were performed in the following anatomical structures:

- *Metabolically active lymphoma lesions*: Up to four representative lymphoma lesions were quantified and analysed per patient. Lesion detection was performed according to the current guidelines and had to be at least 0.9 ml in size and clearly distinguishable from surrounding structures [12].
- *Unaffected healthy lymph node*: Healthy lymph nodes were selected as a reference for baseline metabolic activity and one representative lymph node was quantified for each patient.
- *Reference organs*: SUV uptake of liver and spleen was quantified as reference organs to facilitate the evaluation of metabolic activity in standard tissues.
- *Bone marrow*: VOIs were placed within specific vertebral bodies, including the 10th thoracic vertebra, the 3rd lumbar vertebra, and the sacrum, to assess bone marrow uptake.
- *Muscle*: VOIs were placed within the gluteal muscle to asses the muscle uptake.
- *Blood pool*: Metabolic activity within the blood pool was measured using a VOI placed in the right ventricle of the heart to serve as an internal reference.
- *Brain parenchyma*: Brain grey matter was quantified using a semi-automatic segmentation tool, with a basal ganglia focused threshold to ensure clear delineation of the white matter, using proprietary software (Affinity Hybrid Viewer v.3.0.5, Hermes Medical Solutions, Sweden). The VOI was defined in the clinical PET scan, and its size and position were transferred to the late and ultra-late scans after co-registration of all three scans.

The following semi-quantitative parameters were obtained and specific ratios were calculated to compare metabolic activity across different tissues:

- *SUVmean*: mean SUV calculated from all voxels within the VOI by an isocontour threshold of 50% of the lesion’s maximum SUV.
- *TBR*: Ratio of SUVmean of lymphoma/blood pool.
- *RI*: Retention index was defined as the percentage change in lesion SUVmean between two time points: RI = ((SUVdelay – SUVearly)/SUVearly) × 100 [30].

Image analysis was performed using clinically validated Siemens SyngoVia v.8.0 software (Siemens Healthineers). Routine PET/CT and study-related late/ultra-late scans were jointly reviewed in consensus by board-certified nuclear medicine and radiology experts with at least 5 years of experience in hybrid imaging.

### Qualitative image analysis

Two board-certified specialists independently assessed all late- and ultra-late PET scans. Rater 1 (JV, nuclear medicine, 5 years) and rater 2 (CR, radiology, 8 years) evaluated image quality, lesion conspicuity, and noise using a structured Likert scale (Table 1). Assessments were blinded and each late scan was compared side-by-side with the standard 1 h p.i. PET as reference.

**Table 1:** Qualitative assessment of late PET examinations

SCORE	Image Quality	Lesion Conspicuity	Image Noise
5	state-of-the-art quality	well-defined	near-imperceptible noise
4	superior to the average	fairly defined	lower than regular image of daily practice
3	regular quality of daily practice	hazy, recognizable	similar to regular image of daily practice
2	barely diagnostic	ill-defined, impairing diagnostic confidence	increased noise, slightly worse than regular image of daily practice
1	non-diagnostic	un-recognizable	excessive noise

## Statistics

To systematically assess differences in [^18^F]-FDG uptake over time across anatomical sites and variables, time–activity curves were modelled using mixed-effects regression. To capture all trajectory characteristics detectable with three time points, both linear and quadratic time components were included. Repeated measures within individuals were accounted for by modelling random intercepts throughout. Depending on the most plausible distribution of residuals, either standard multiple linear mixed-effects regressions (with polynomial time terms) or Gamma generalised linear mixed-effects regressions with a log link were applied. The fixed-effect trajectory components were estimated using:

Linear mixed models:

$$Y=Intercept+LIN+QUA+e$$

Gamma mixed models:

$$\ln(Y)=Intercept+LIN+QUA$$

where LIN represents identity-coded time post-injection (0 for 1 h p.i., 2 for 3 h p.i., and 5 for 6 h p.i. to preserve temporal spacing), and QUA corresponds to LIN².

Where subgroup differences (moderators) were examined, interaction terms were added, yielding:

Linear mixed models:

$$\begin{array}{c}Y=Intercept+LIN+QUA+\lbrack Moderator\rbrack+LIN\\\times\lbrack Moderator\rbrack+QUA\times\lbrack Moderator\rbrack+e\end{array}$$

Gamma mixed models:

$$\begin{array}{c}Y=Intercept+LIN+QUA+\lbrack Moderator\rbrack+LIN\\\times\lbrack Moderator\rbrack+QUA\times\lbrack Moderator\rbrack\end{array}$$

Coefficients were tested using *t* statistics in linear mixed models and Wald tests in Gamma models. A test-wise significance level of α = 0.05 was applied.

## Results

### Patient cohort

Treatment-naïve patients with histologically confirmed lymphoma (*n* = 15) underwent the standard of care PET/CT scan at 1 h p.i. and the study-related late PET/CT scan 3 h p.i. 14/15 patients opted for the ultra-late PET/CT scan at 6 h p.i. Male sex was predominant in this trial with 9 males and 6 females participating. The average patient age was 56.9 ± 20.4 years (range 19–83 y) with an average body weight of 81.8 ± 18.5 kg and body size of 174 ± 8.5 cm respectively, a BMI of 27.0 ± 5.9 kg/m² (range 18.7–41.6). The injected activity was 3.03 ± 0.34 MBq of [^18^F]FDG per kg (246.73 ± 62.50 MBq). The sample included 4 patients with Hodgkin’s and 11 patients with non-Hodgkin’s lymphoma (Table 2). In total, 9 patients were classified as high-grade and 6 patients as low-grade lymphoma, with details for each patient presented in Table 2. In total, 42 lymphoma lesions were quantified at 1 h p.i., 42 lesions at the late scan 3 h p.i., and 38 lesions in the ultra-late PET scan at 6 h p.i.

**Table 2:** Patient characteristics

Patient ID	Sex	Age in years	Histology	Grade	Ann Arbor
1	Female	55	Follicular lymphoma	Low	IIIb
2	Male	74	Follicular lymphoma	Low	IIa
3	Female	29	Castleman disease	Low	-
4	Male	64	Mantle cell lymphoma	Low	IAE
5	Female	19	DLBCL, GCB	High	IIISA
6	Female	56	Hodgkin lymphoma	High	IIA
7	Male	21	Hodgkin lymphoma	High	III S
8	Male	47	DLBCL, GCB	High	IIIAE
9	Female	74	Extranodal marginal zone lymphoma of the lung	Low	IVA
10	Male	69	Peripheral T-cell lymphoma	Low	IA
11	Male	62	Hodgkin lymphoma	High	IV
12	Female	83	DLBCL	High	IV
13	Male	74	DLBCL	High	IIE
14	Male	77	DLBCL	High	IV
15	Male	50	Hodgkin lymphoma	High	II

* DLBCL* Diffuse Large B-Cell Lymphoma; *GCB* Germinal Center B-Cell

### Image quality

Late PET scans 3 h p.i. presented an overall high visual image quality (Likert score: 4, range: 4–5) compared to the reference scan at 1 h p.i. with fairly defined lesion conspicuity (Likert score median: 4, range: 3–5, *p* > 0.05) with only slightly increased image noise (Likert score median: 4, range: 4–5, *p* > 0.05). In the ultra-late scans 6 h p.i. image noise increased (Likert score: 3, range: 3–4, *p* < 0.05) along with a decrease in image quality (Likert score: 3–4, range: 2–4, *p* > 0.05) but with persistent fairly defined lesion conspicuity (Likert score 3, range: 3–5, *p* > 0.05).

### [^18^F]FDG pharmacokinetics

**Fig. 2:**
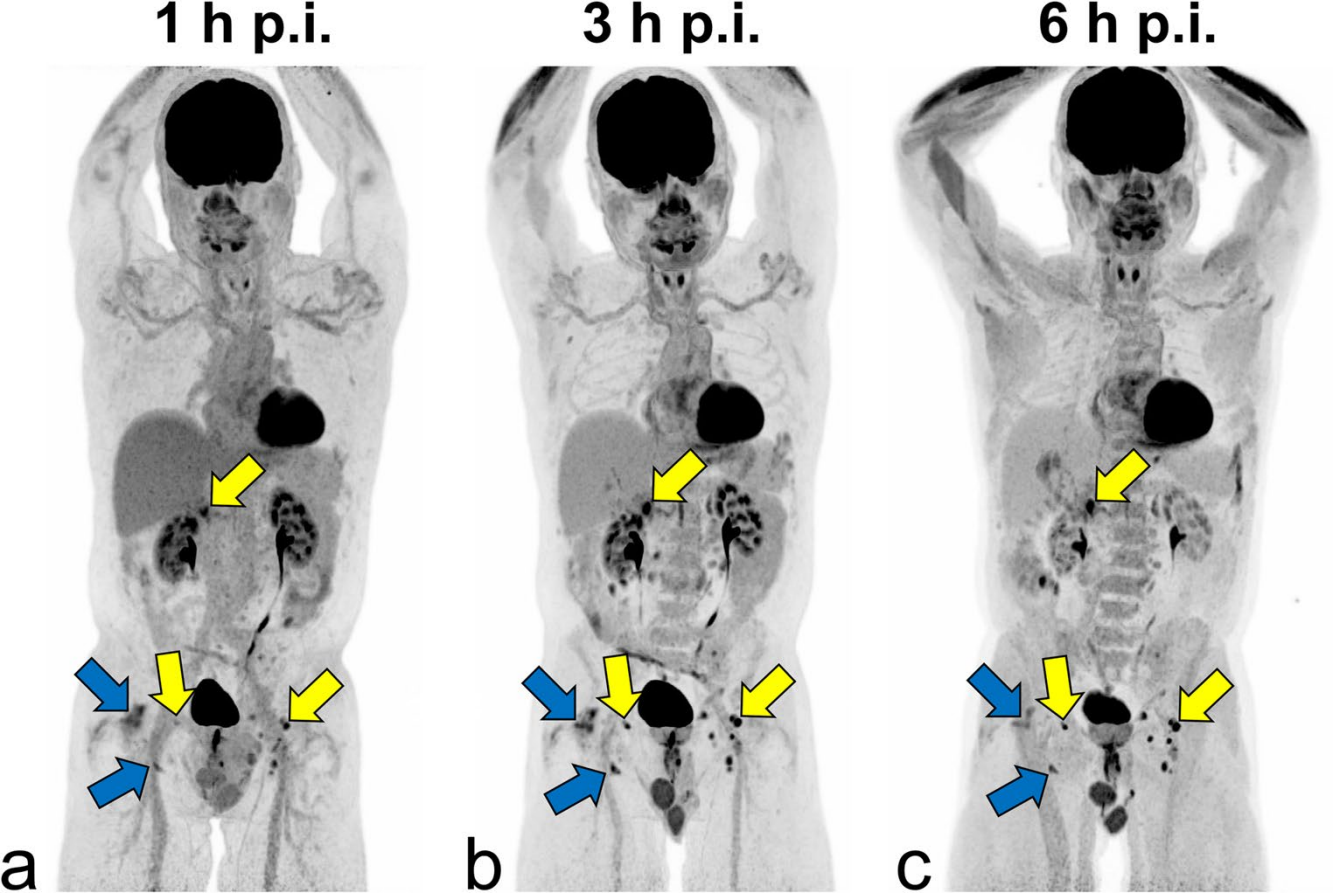
Maximum intensity projections of [¹⁸F]FDG PET/CT (3 MBq/kg) in a 74-year-old patient with newly diagnosed follicular lymphoma (grade 1–2), acquired at 1 h (**a**), 3 h (**b**), and 6 h (**c**) p.i. Multiple pathologically enlarged lymph nodes are observed in the retroperitoneal and bilateral inguinal regions. At 1 h p.i., some lymph nodes show moderately increased metabolic activity (yellow arrows), while others appear metabolically inactive (blue arrows). Delayed imaging at 3 h and 6 h p.i. reveals progressive [^18^F]FDG accumulation in all marked lymph nodes with improved TBR, alongside decreasing uptake in parenchymal organs and the blood pool. Additionally, increased [^18^F]FDG uptake in the bone marrow and skeletal muscles is noted on the late-phase scans

**Fig. 3:**
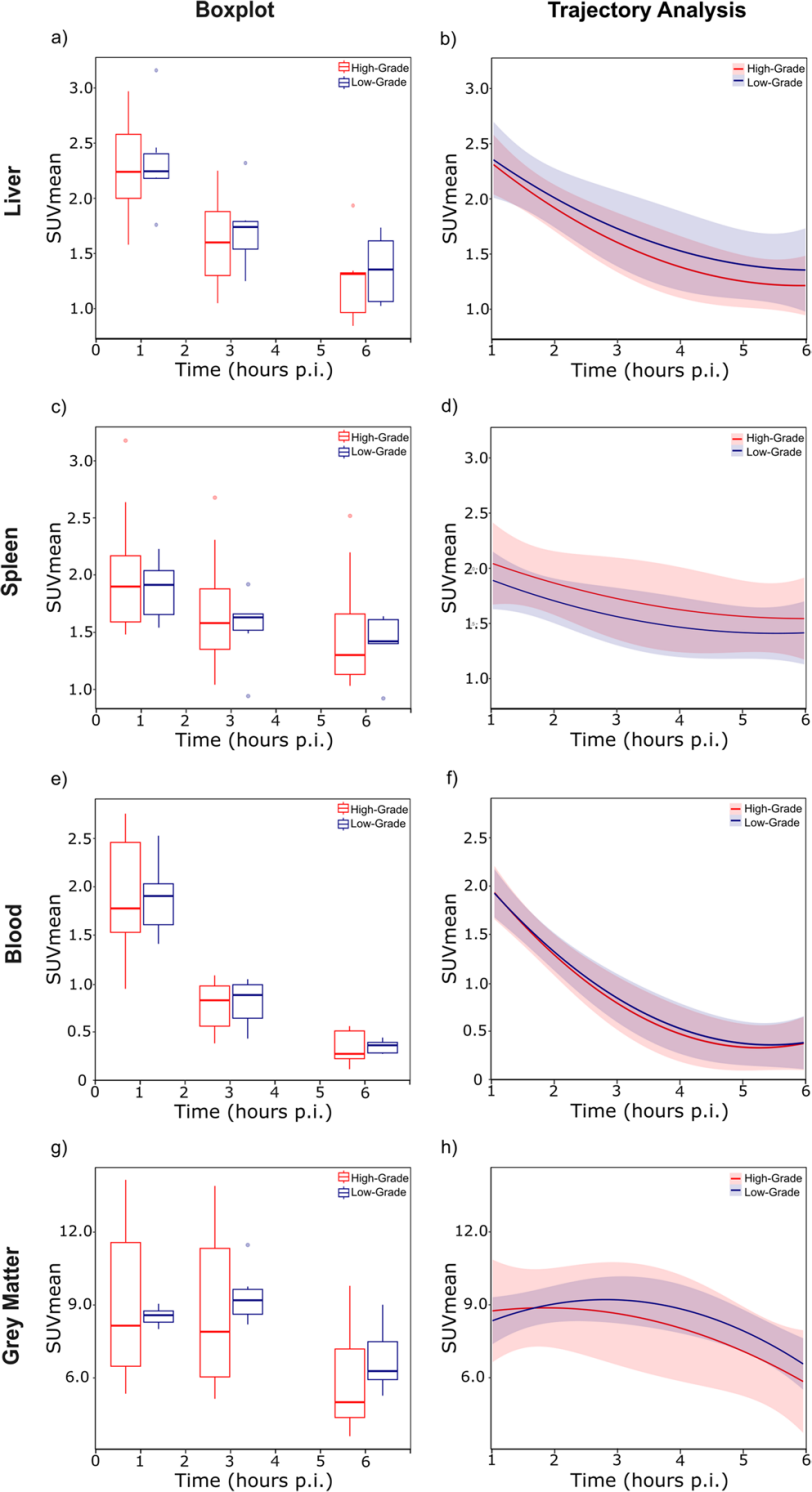
[^18^F]FDG Radiotracer kinetics, expressed as SUVmean uptake, are shown for routine (1 h p.i.), late (3 h p.i.), and ultra-late (6 h p.i.) PET scans. Data are visualized as boxplots (**a**,** c**,** e**,** g**) and time-activity curve fits (**b**,** d**,** f**,** h**) with linear and quadratic components. Organ-specific kinetics are displayed for liver parenchyma (**a**, **b**), spleen (**c**,** d**), blood pool (**e**,** f**), and brain grey matter (**g**,** h**), stratified by high-grade (red) and low-grade (blue) lymphoma

Reference organs, including the liver, spleen, and blood pool showed a significant decline in [^18^F]FDG uptake at 3 and 6 h p.i. compared with 1 h p.i. scan as shown in a representative scan in Fig. 2. The data are summarised in Fig. 3; Table 3. In contrast, [¹⁸F]FDG uptake in the bone marrow and gluteal muscles increased almost linearly over time. Human brain grey matter demonstrated a parabolic uptake pattern, with peak [^18^F]FDG uptake at 3 h p.i. followed by a significant decline at 6 h p.i.

**Table 3:** [^18^F]FDG uptake in lymphoma, benign lymph nodes and reference organs quantified with SUVmean at 1 h, 3 h and 6 h p.i

Organs	Time-point			*p*-value		
	1 h p.i.	3 h p.i.	6 h p.i.	1–3 h p.i.	1–6 h p.i.	2–6 h p.i.
Lymphoma all	7.7 ± 4.8	9.4 ± 6.5	7.7 ± 4.5	0.180	0.850	0.137
Low-grade	8.7 ± 7.0	11.3 ± 10.3	6.1 ± 5.1	0.465	0.395	0.199
High-grad	7.4 ± 3.8	8.7 ± 4.5	7.9 ± 4.3	0.219	0.601	0.492
Benign lymph nodes	0.9 ± 0.3	0.8 ± 0.5	0.8 ± 0.4	0.654	0.359	0.725
Liver	2.4 ± 0.4	1.7 ± 0.4	1.3 ± 0.3	**< 0.001**	**< 0.001**	**0.006**
Spleen	2.0 ± 0.5	1.7 ± 0.5	1.5 ± 0.5	0.064	**0.008**	0.343
Blood pool	2.0 ± 0.5	0.8 ± 0.3	0.4 ± 0.1	**< 0.001**	**< 0.001**	**< 0.001**
Muscle*	0.6 ± 0.1	0.8 ± 0.3	1.2 ± 0.4	**0.007**	**< 0.001**	**0.003**
Bone marrow	1.5 ± 0.6	1.8 ± 0.9	2.0 ± 1.0	0.206	0.060	0.573
Grey matter	8.8 ± 2.5	9.0 ± 2.6	6.3 ± 2.0	0.772	**0.008**	**0.004**

The SUVmean values that differ significantly between two time-points in the two-sided t-test are marked in bold. *Quantified in gluteal muscles

**Fig. 4:**
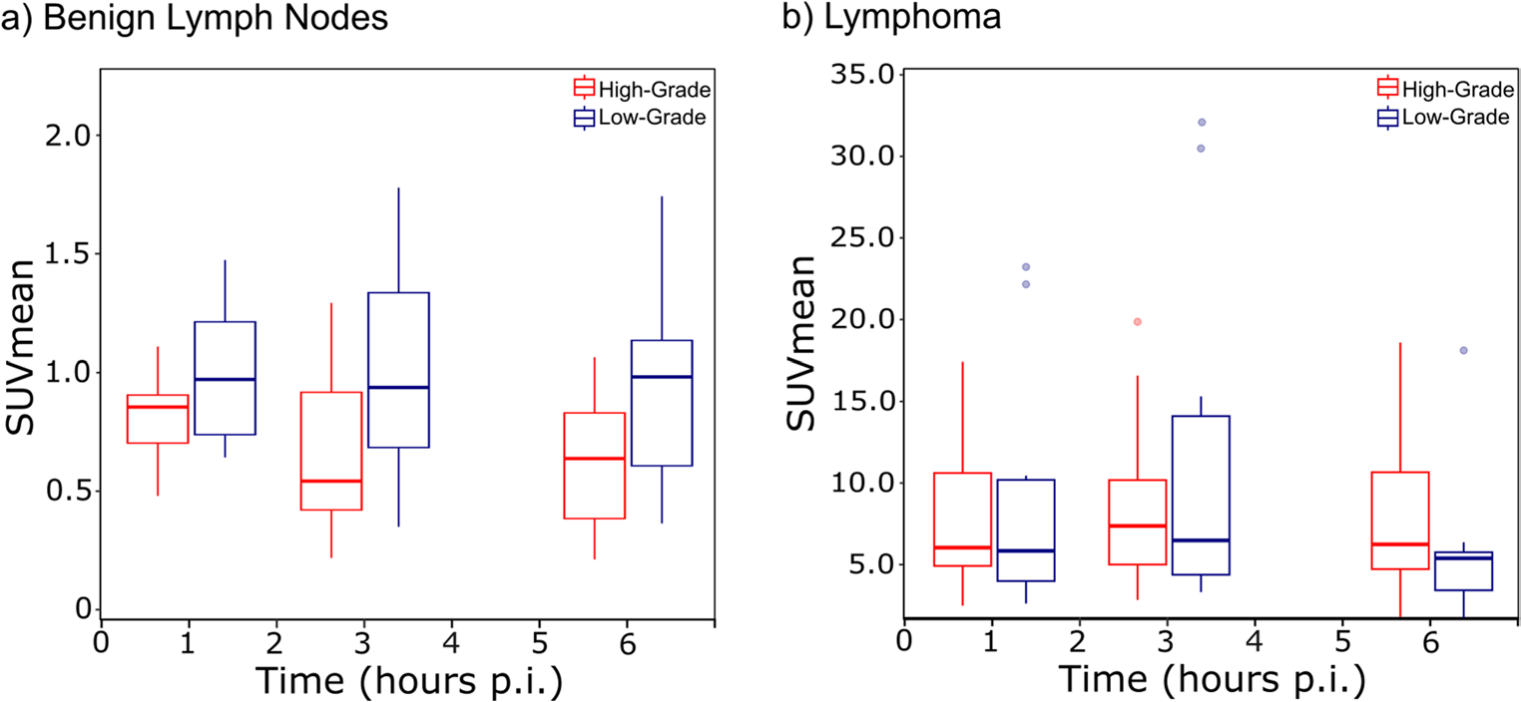
[^18^F]FDG uptake, quantified as SUVmean, in benign lymph nodes (**a**) and lymphoma (**b**) at the time-points of the routine clinical scan (1 h p.i.), late PET scan (3 h p.i.), and ultra-late PET scan (6 h p.i.). The mean SUV values for high-grade and low-grade lymphoma are plotted in red and blue, respectively

**Fig. 5:**
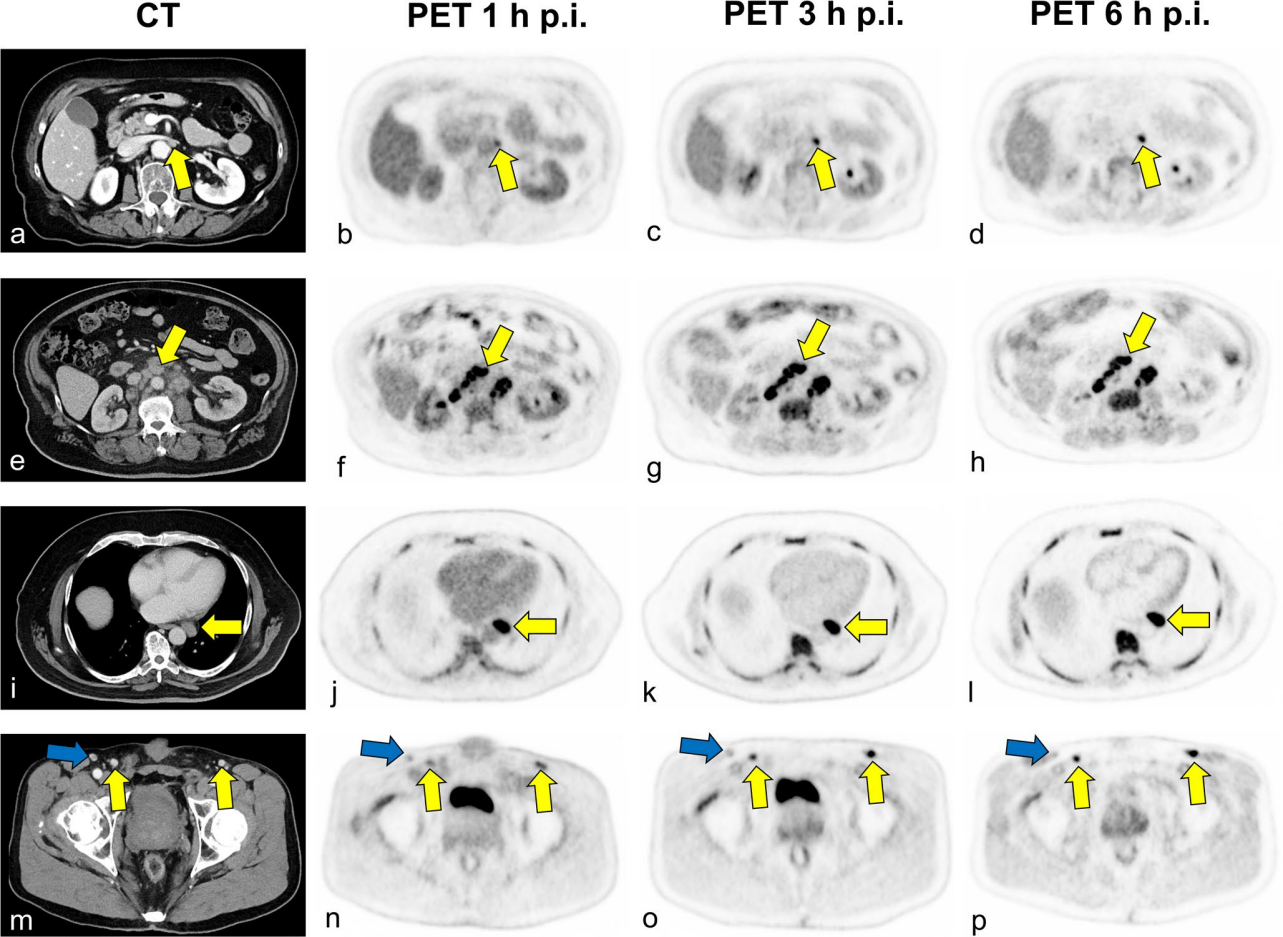
Representative cases demonstrating delayed [^18^F]FDG PET/CT imaging in patients with lymphoma. **a–d**: 83-year-old female patient with histologically confirmed DLBCL, grade 4. A pathologically enlarged lymph node in the left para-aortic region at the level of the left renal vein confluence (**a**) shows increased [^18^F]FDG uptake at 1 h p.i. (**b**), with further rising uptake at 3 h (**c**) and 6 h (**d**) p.i. (yellow arrows). **e–l**: 62-year-old male patient with histologically confirmed Hodgkin lymphoma, grade 4. Enlarged lymph nodes in the upper retroperitoneum (**e–h**) and paracardial region (**i–l**) show intense [^18^F]FDG uptake at 1 h p.i. (**f** and **j**, yellow arrows) and demonstrate further increase in tracer accumulation in delayed scans at 3 h and 6 h p.i. (**g–h** and **K–L**, yellow arrows). **m–p**: 4-year-old male patient with newly diagnosed follicular lymphoma (grade 1–2) and multiple pathologically enlarged lymph nodes in the bilateral inguinal regions **(m)**. Some lymph nodes exhibit moderately increased [^18^F]FDG uptake (yellow arrow) with progressive uptake observed in delayed imaging **(n-p)**, while others appear metabolically inactive at all time points (blue arrows)

Lymphoma exhibited significantly higher [^18^F]FDG uptake than benign lymph nodes, following parabolic kinetics and peaking at 3 h p.i. as shown in Fig. 3; Table 3. In contrast, benign lymph nodes showed an almost stable [^18^F]FDG uptake over time as plotted in detail in Fig. 4 with a representative case in Fig. 5. Lymphoma lesions also had a significantly higher retention index (RI) compared to benign lymph nodes (1–3 h p.i.: 19.4% vs. −14.4%, *p* < 0.001; 1–6 h p.i.: 11.4% vs. −16.9%; *p* = 0.03). The TBR of the lymphoma significantly increased over time, from 4.1 ± 2.5 at 1 h p.i. to 12.5 ± 7.3 at 3 h p.i. and 23.9 ± 15.2 at 6 h p.i. (*p* < 0.001) as shown in Supplementary Fig. 1.

Bone marrow infiltration was histologically confirmed in three patients, but SUVmean did not differ significantly at any time-point (SUVmean values are shown in Supplementary Fig. 2).

### Differentiation of lymphoma subtypes

**Fig. 6:**
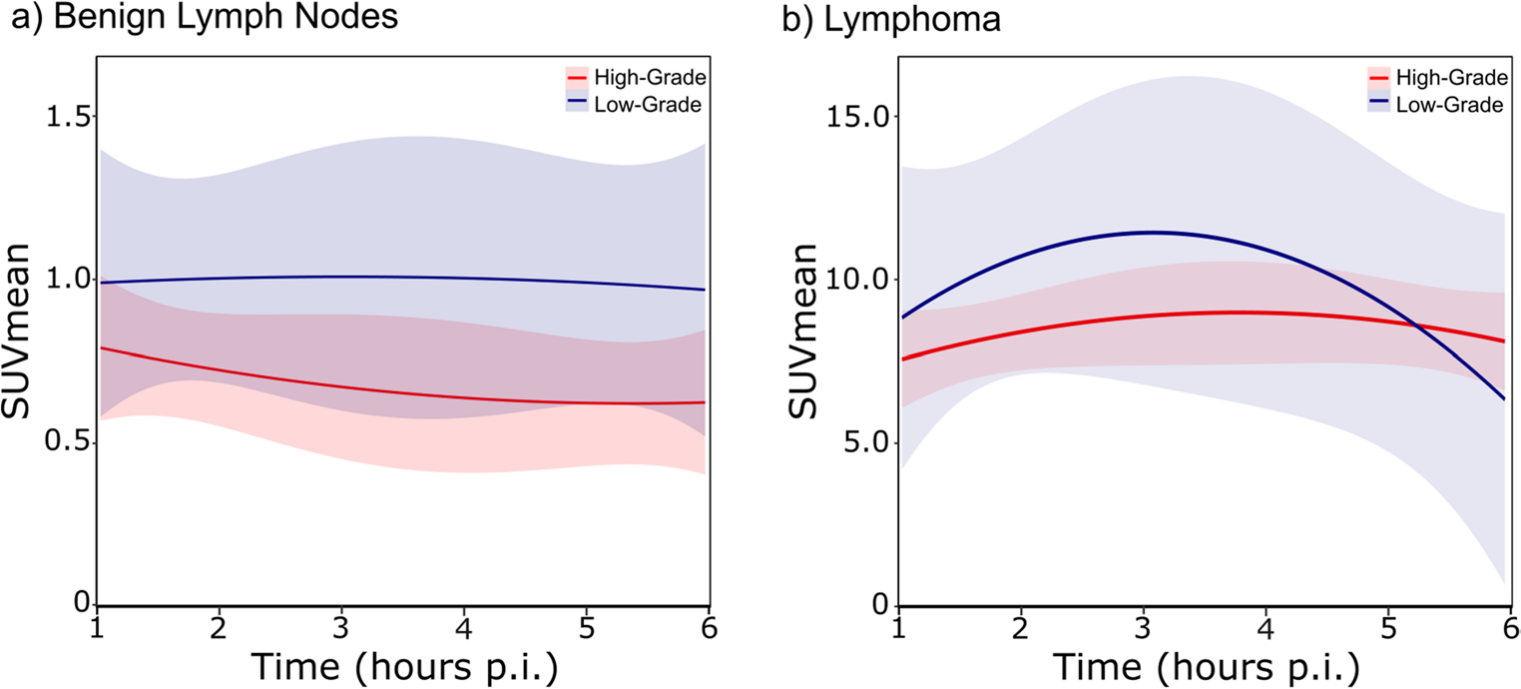
[^18^F]FDG time-activity curves displaying SUVmean values for high-grade (red) and low-grade (blue) lymphoma, with linear and quadratic trajectory components (**a**). Panel (**b**) shows the corresponding uptake curves for non-involved lymph nodes from patients with high-grade (red) and low-grade (blue) lymphoma

The [^18^F]FDG kinetics revealed significant differences in uptake patterns between low-grade and high-grade lymphoma, as illustrated in Fig. 6 and Supplementary Fig. 1b. Measurements for low-grade lymphoma were most consistent with a parabolic uptake curve along an overall increase, with SUVmean peaking at 3 h p.i. In contrast, high-grade lymphoma showed a more pronounced linear increase and less pronounced parabolicity in SUVmean from 1 h to 6 h p.i. (OR for linear component high-grade lymphoma vs. low-grade lymphoma: 1.06; 95% CI: 1.05–1.07; *p* < 0.001, OR for parabolic component high-grade lymphoma vs. low-grade lymphoma: 0.99; 95% CI: 0.98–1.00; *p* = 0.007). However, SUVmean at individual time-points did not significantly differentiate between low- and high-grade lymphoma (1 h p.i.: 8.7 ± 7.0 vs. 7.4 ± 3.8; 3 h p.i.: 11.3 ± 10.3 vs. 8.7 ± 4.5; 6 h p.i.: 6.1 ± 5.1 vs. 7.9 ± 4.3), as shown in Figs. 4 and 6.

### Follow up

Of the 15 enrolled patients, 10 underwent in-house follow-up imaging and 1 was followed up externally within 6 ± 3 months after the study. One patient passed away and data were not available for the remaining 3 patients. 9 patients received guideline-based systemic therapy, all achieving complete remission. Meanwhile 2 patients managed on the watch-and-wait strategy remained clinically stable.

In histology and follow-up imaging, 1/11 patient was upstaged from PET/CT stage IIA to stage IV due to immunohistochemically confirmed bone marrow infiltration which was undetected in the initial [^18^F]FDG PET 1 h p.i. and revealed no increased uptake in 3 h p.i. and 6 h p.i. PET scan.

### Radiation exposure

The study-specific excess radiation exposure for ultra-low-dose CT attenuation correction of the 106 cm PET field of view (FOV) was 0.26 ± 0 mSv for each late and ultra-late PET/CT scan.

## Discussion

PET/CT imaging with [^18^F]FDG is the state-of-the-art method in lymphoma staging. However, sensitivity is limited in certain scenarios, such as distinguishing lymphoma from inflammatory lymph nodes and identifying lymphoma infiltration in organs with high glucose metabolism like the spleen or kidney. These limitations have significant implications for the Ann Arbor classification and subsequent treatment decisions.

As delayed whole-body [^18^F]FDG imaging in lymphoma is largely unexplored, our prospective study aimed to investigate the extended [^18^F]FDG pharmacokinetic and to evaluate the feasibility and diagnostic value of ultra-late [^18^F]FDG PET/CT imaging up to 6 h p.i. Additional insights into [^18^F]FDG kinetics may improve lymphoma detection, distinguishing from inflammatory lymph nodes and other tissues with high [^18^F]FDG uptake and support differentiation between high- and low-grade lymphoma subtypes.

This prospective trial provides new insights into whole-body [^18^F]FDG kinetics in lymphoma patients through multi time-point whole-body [^18^F]FDG PET/CT scans at 1 h, 3 h and 6 h p.i. enabled by a LAFOV system. Evidence on late total-body [^18^F]FDG PET/CT imaging up to 6 h p.i. in lymphoma remains limited and this study provides an initial prospective assessment in this emerging field. The markedly increased sensitivity of LAFOV PET - up to tenfold higher than conventional systems - allows diagnostic imaging across several tracer half-lives without requiring increased injected activity [26, 31].

We observed distinct patterns of [^18^F]FDG uptake between high- and low-grade lymphoma and benign lymph nodes. High-grade lymphoma revealed an almost linear increase in [^18^F]FDG uptake over time, peaking at 6 h p.i. By contrast, low-grade lymphoma exhibited a parabolic uptake pattern, peaking at 3 h p.i. These differences in uptake kinetic were significant between high- and low-grade lymphoma, although the absolute SUV values at individual time-points did not differ significantly. These findings are consistent with the rare literature data on solid tumours, such as sarcomas, where high-grade lesions exhibited a significantly delayed activity peak (about 4 h p.i.) compared to low-grade sarcomas (within 1 h p.i.) [32]. Benign lymph nodes, in contrast, revealed an almost stable [^18^F]FDG uptake over time. Nevertheless, it remains unclear whether inflammatory lymph nodes or those affected by sarcoid-like reaction exhibit a similarly stable [^18^F]FDG uptake pattern.

Differences in kinetic patterns could not only serve to distinguish between benign tissue and lymphoma, but could also be useful as imaging biomarkers, providing additional information on lymphoma subtypes, metabolism and ultimately risk stratification. Sequential ultra-late PET imaging could provide important information about the biological characteristics of lymphoma and would be of high clinical interest, especially in cases of lesions inaccessible to biopsy. These initial results, of course, need to be confirmed in multicentre studies with larger sample sizes.

In addition, the tumour-to-background ratio increased significantly at 3 and 6 h p.i., which may improve the detectability of lymphoma lesions in highly [^18^F]FDG avid organs as the spleen and kidneys. These findings are consistent with previous studies emphasizing the role of prolonged uptake times up to 3 h p.i. in enhancing lesion contrast [14, 22]. Unfortunately, no patient in this cohort showed such organ involvement, so this hypothesis could not be validated.

Beyond its potential to improve lymphoma staging, the prolonged [^18^F]FDG kinetics observed in this study provide new insights into the underlying glucose metabolism of these malignancies. High-grade lymphoma demonstrated a continuous increase in [^18^F]FDG activity concentration up to 6 h p.i., a pattern analogous to that reported for several solid tumours [33]. In contrast, low-grade lymphoma exhibited a parabolic uptake pattern with a peak at approximately 3 h p.i., followed by a subsequent decline. Comparable parabolic time-activity curves -with peaks occurring around 4 h p.i.- have also been described for lymph node metastases of non-small-cell lung cancer and for high-grade sarcoma. These findings support the hypothesis that delayed [^18^F]FDG release may also occur in low-grade lymphoma. This challenges the classical concept of irreversible intracellular trapping in malignant tissue [32, 34]. Studies using late PET acquisitions with a limited FOV have previously suggested that a delayed radioactivity release, for example through [^18^F]FDG efflux, could be a plausible mechanism [32].

One potential explanation might be related to variabilities in the glucose-6-phosphate transporter (G6PT) density and glucose-6-phosphatase-β (G6Pase-β) activity, which was shown to correlate with [^18^F]FDG efflux rate [35]. G6PT was shown to translocate the intracellularly trapped [^18^F]FDG-6-phosphate into the endoplasmic reticulum (ER), where it can be hydrolysed by G6Pase-β [36–39]. The ER thus represents both a potential site of radioactivity accumulation and a pathway for [^18^F]FDG efflux [36, 40]. While hydrolysis of [^18^F]FDG-6-phosphate is usually considered negligible within the clinical PET/CT timeframe (60–90 min) [41–43], distinct kinetic profiles in late and ultra-late PET might potentially be affected by G6Pase-β activity. The sustained [^18^F]FDG uptake observed in high-grade lymphoma up to 6 h p.i. may suggest lower G6Pase-β activity and reduced efflux of [^18^F]FDG into the extracellular space in high-grade subtypes such as Hodgkin lymphoma or DLBCL. Conversely, the decrease in SUVmean observed in low-grade lymphoma between 3 and 6 h p.i. may reflect residual G6Pase-β activity and subsequent efflux of dephosphorylated [^18^F]FDG-6-phosphate [42, 43]. This hypothesis is supported by Lodge et al.‘s findings, which revealed a quantifiable dephosphorylation rate (k₄) of 4.76 × 10⁻⁵ s⁻¹ in high-grade sarcomas through dynamic imaging and Patlak modelling [32].

Given the complexity of metabolic reprogramming during malignant transformation, multiple factors are likely to contribute to these differing kinetic profiles. A further possible explanation may be an earlier saturation of the anaerobic glycolytic pathway in low-grade lymphoma, resulting in intracellular accumulation of unphosphorylated [^18^F]FDG and consequently, a peak in tracer uptake at 3 h p.i., followed by subsequent efflux of [^18^F]FDG. In contrast, high-grade lymphoma may rely more heavily on anaerobic metabolic mechanisms and are therefore able to retain the glucose analogue more effectively than their low-grade counterparts [44].

The presence of a non-negligible [^18^F]FDG efflux in delayed PET imaging has important implications for compartment modelling in dynamic PET, particularly when applying the Patlak analysis, which assumes irreversible trapping (k₄ = 0) [45, 46]. Consequently, accurate estimation of the metabolic rate of lymphoma in late acquisitions would require explicit consideration of k₄, for example through an extended Patlak approach [32, 45].

The parabolic [^18^F]FDG uptake pattern, characterized by a marked decrease in activity between 3 and 6 h p.i. was also observed in brain grey matter and is consistent with the findings of Spence et al., who attributed the phenomenon to high G6Pase activity [34]. The significantly lower [^18^F]FDG activity in the grey matter on ultra-late PET scans may improve the contrast between cerebral malignancies and the surrounding unaffected parenchyma [34]. The diagnostic value of late PET imaging should be further investigated in studies focusing on CNS lymphoma.

Conventional PET/CT imaging protocols typically utilize acquisition times at 60–90 min p.i., balancing tracer uptake with image quality [12]. Our findings demonstrate that ultra-late imaging - enabled by LAFOV PET systems - can extend the diagnostic window to at least 6 h p.i. while maintaining high image quality, albeit still requiring a 30 min long acquisition time. Further scan time reduction can be achieved using the recently introduced ultra-high sensitivity (UHS) mode of the Biograph Vision Quadra, which leverages the full acceptance angle, enabling up to 50% shorter acquisitions and thereby potentially enhancing the clinical feasibility of ultra-late imaging protocols [47, 48].

Repetitive imaging, especially in young oncology patients, raises significant concerns regarding radiation exposure. In conventional PET systems, CT-based attenuation correction contributes additional radiation. However, the implementation of our ultra-low-dose CT protocol (140 kV, 6 mAs, tin filter) reduced the effective dose to 0.26 mSv per scan. To further reduce radiation exposure, attenuation correction approaches without the need of CT have been proposed leveraging the increased number of scintillation crystals and the intrinsic radiation of LSO to generate transmission based attenuation maps [49, 50]. These technical advances may enable late PET acquisitions in paediatric patients without additional radiation to sort out extranodal lesions, which are more prevalent in children [51–53].

## Limitations

The primary limitations of our study include its single-centre design, small sample size and the use of only two additional scan time-points to minimise patient discomfort, so that a granular description of tracer kinetics remains limited.

The heterogeneous sample reflects the range of lymphomas typically encountered in clinical practice. However, due to the limited sample size, only comparisons between high- and low-grade lymphomas were statistically meaningful. Consequently, the results should be interpreted as indicative trends rather definitive conclusions about specific subtypes.

Patients with a clinical indication for PET/CT were enrolled consecutively, however the demanding protocol may have introduced selection bias by favouring the participation of patients in sufficiently stable clinical condition, however there was no underrepresentation of patients suffering from high-grade lymphoma.

Only three patients had confirmed bone marrow infiltration, limiting statistical power for subanalyses of [^18^F]FDG kinetics in bone marrow lesions. We observed a significant increase in muscular uptake up to 6 h p.i., which in principle reduces the amount of tracer available for tumour uptake. However, dosimetry analysis demonstrated that global muscle uptake accounted for approximately 0.5% of the injected dose, meaning its impact on SUV measurements in lymphoma lesions can be considered as minimal [54]. Moreover, any potential impairment in the detectability of muscular lesions at 6 h p.i. could not be assessed, as no extranodal muscular involvement was present in our cohort.

Ground truth was determined by clinical PET/CT, histological analysis from lymph nodes and bone marrow and follow-up data. In accordance with the current ESMO guidelines [3, 6, 7], only single lymph node biopsies - with excision often performed before the PET scan - and bone marrow biopsies were obtained, which were assumed to be representative of all PET positive lesions. Consequently, a one-to-one correlation between histology and PET from the same lesion was not feasible.

Ultimately, the clinical added value of late and ultra-late PET scans was not the primary objective of this observational study. The main aim was to gain initial experience in interpreting ultra-late PET imaging in this novel setting.

## Conclusions

Ultra-late whole-body [^18^F]FDG PET/CT imaging using a LAFOV scanner provides high-quality imaging up to 6 h p.i. Low- and high-grade lymphoma revealed distinct [^18^F]FDG kinetics. High-grade subtypes demonstrated a continuous increase in [^18^F]FDG uptake up to 6 h p.i., whereas low-grade subtypes peaked at 3 h p.i. followed by a decline.

Consequently, a three-point ultra-late PET protocol (1, 3, and 6 h p.i.) may provide valuable metabolic information and may assist in the differentiation of high- from low-grade lymphoma in cases where lesions are inaccessible by biopsy. For staging purposes, imaging at 3 h p.i. improves the tumour-to-background ratio significantly.

## Supplementary Information

Below is the link to the electronic supplementary material.

Supplementary figure 7 Boxplots of the tumour-to-background ratio (TBR), calculated as the quotient of SUVmean of lymphoma and blood pool activity in the right atrial lumen, at 1 h, 3 h, and 6 h p.i. in patients with high-grade (red) and low-grade (blue) lymphoma. TBR increased significantly between 1 h and 6 h p.i., (**a**) following a linear trajectory in high-grade and a parabolic trajectory in low-grade lymphoma (**b**). (PNG 636 KB)

High Resolution Image (TIF 771 KB)

Supplementary figure 8 SUVmean values of bone marrow at routine (1 h p.i.), late (3 h p.i.), and ultra-late (6 h p.i.) PET scans. [^18^F]FDG uptake increased in both non-affected (**a**) and infiltrated (**b**) bone marrow between 1 h and 6 h p.i., with higher but very heterogeneous uptake changes in cases of bone marrow involvement.(PNG 816 KB)

High Resolution Image (TIF 739 KB)

## Data Availability

The datasets generated and analysed during the current study are available from the corresponding author on reasonable request.
