# Supplementary figures and images for: Exploring extended [18F]FDG kinetics in lymphoma with ultra-late LAFOV-PET/CT
Source: Eur J Nucl Med Mol Imaging. 2026 Feb 11;53(7):4494–505. doi: 10.1007/s00259-026-07774-w (PMC13197291; doi:10.1007/s00259-026-07774-w)

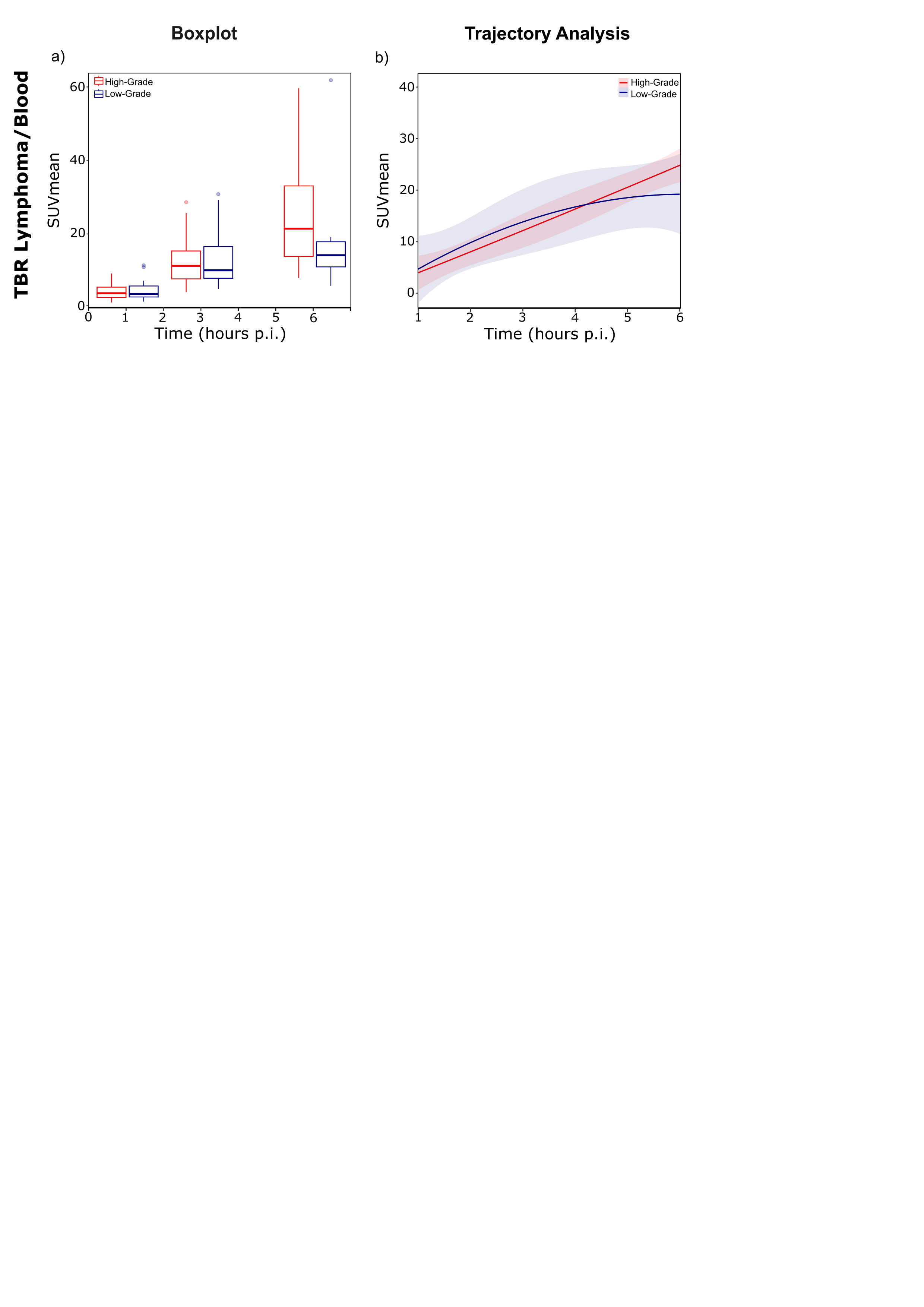

Supplement: Supplementary file 2 — High Resolution Image (TIF 771 KB) [file 259_2026_7774_MOESM1_ESM.tiff]

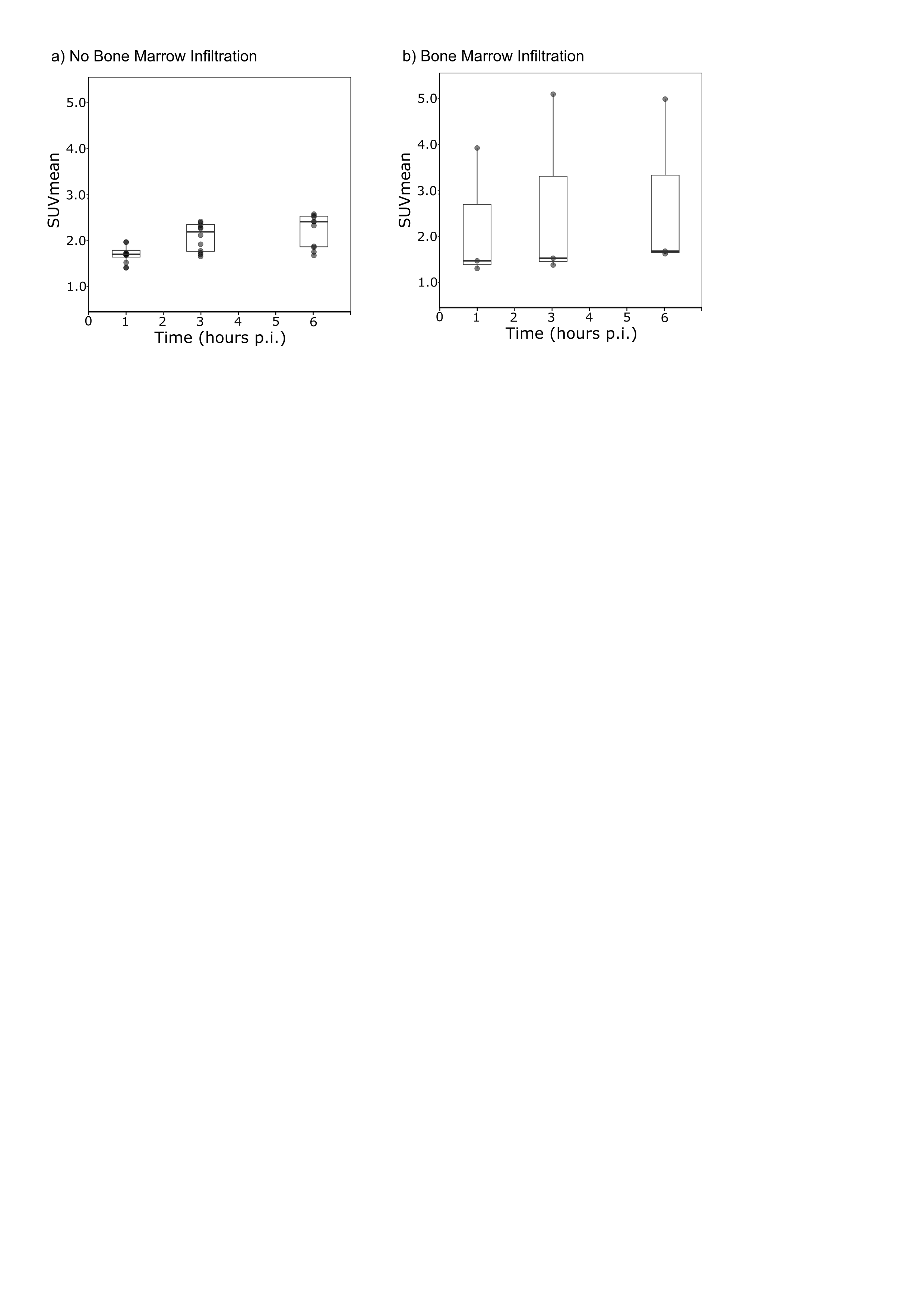

Supplement: Supplementary file 4 — High Resolution Image (TIF 739 KB) [file 259_2026_7774_MOESM2_ESM.tiff]
